# Performance of a glucose-reactive enzyme-based biofuel cell system for biomedical applications

**DOI:** 10.1038/s41598-019-47392-1

**Published:** 2019-07-26

**Authors:** Won-Yong Jeon, Jung-Hwan Lee, Khandmaa Dashnyam, Young-Bong Choi, Tae-Hyun Kim, Hae-Hyoung Lee, Hae-Won Kim, Hyug-Han Kim

**Affiliations:** 1 Department of Chemistry, College of Natural Science, Dankook University, Chungnam, Cheonan 31116 Republic of Korea; 2 Institute of Tissue Regeneration Engineering (ITREN), Dankook University, Chungnam, Cheonan 31116 Republic of Korea; 3 Department of Nanobiomedical Science & BK21 PLUS NBM Global Research Center for Regenerative Medicine, Dankook University, Chungnam, Cheonan 31116 Republic of Korea; 4 UCL Eastman-Korea Dental Medicine Innovation Centre, Dankook University, Chungnam, Cheonan 31116 Republic of Korea; 5 Department of Biomaterials Science, College of Dentistry, Dankook University, Chungnam, Cheonan 31116 Republic of Korea

**Keywords:** Biocatalysis, Biomedical materials

## Abstract

A glucose-reactive enzyme-based biofuel cell system (EBFC) was recently introduced in the scientific community for biomedical applications, such as implantable artificial organs and biosensors for drug delivery. Upon direct contact with tissues or organs, an implanted EBFC can exert effects that damage or stimulate intact tissue due to its byproducts or generated electrical cues, which have not been investigated in detail. Here, we perform a fundamental cell culture study using a glucose dehydrogenase (GDH) as an anode enzyme and bilirubin oxidase (BOD) as a cathode enzyme. The fabricated EBFC had power densities of 15.26 to 38.33 nW/cm^2^ depending on the enzyme concentration in media supplemented with 25 mM glucose. Despite the low power density, the GDH-based EBFC showed increases in cell viability (~150%) and cell migration (~90%) with a relatively low inflammatory response. However, glucose oxidase (GOD), which has been used as an EBFC anode enzyme, revealed extreme cytotoxicity (~10%) due to the lethal concentration of H_2_O_2_ byproducts (~1500 µM). Therefore, with its cytocompatibility and cell-stimulating effects, the GDH-based EBFC is considered a promising implantable tool for generating electricity for biomedical applications. Finally, the GDH-based EBFC can be used for introducing electricity during cell culture and the fabrication of organs on a chip and a power source for implantable devices such as biosensors, biopatches, and artificial organs.

## Introduction

Biofuel cells have attracted significant attention after their introduction in the biomedical field^1,2^. A biofuel cell can be classified into two types depending on the electricity-generating source: a microorganism-based microbial fuel cell (MFC) and an enzyme-based biofuel cell (EBFC)^3–5^. Compared to MFCs, which have inherent drawbacks such as microbial contamination and uncontrollable microbial activity, EBFCs have been widely investigated for biomedical applications^6^. EBFCs can have different types of enzymes, such as cellobiose dehydrogenase (CDH), glucose dehydrogenase (GDH), and glucose oxidase (GOD) for the anode and laccase, ascorbate oxidase, copper-containing oxidase, and bilirubin oxidase (BOD) for the cathode^7–13^. Among these, GDH and GOD are commonly used in the biomedical field due to the relatively high glucose concentrations in living tissue compared to those of the other consumed components, which can generate a high current density^14,15^.

Many researchers have focused on increasing the current density and lifetime of glucose-reactive EBFCs and reported optimal mediators and enzyme-immobilizing carriers^14–17^. Recently, those glucose-reactive EBFCs have been implanted inside living species, such as insect, clams, lobsters, rats and even humans, for powering biosensors or pacemakers^18–23^. However, when glucose-reactive EBFCs directly contact tissues or organs inside the human body, they can have effects leading to the damage or stimulation of intact tissue due to their byproducts or generated electrical cues, but this has been investigated *in vitro* only to a limited extent^9^.

When EBFCs are utilized in cell culture or an implanted system in the living body, the biological effects including safety might differ depending on the enzyme types, amounts and absolute current density. Thus, the *in vitro* biological effects have been performed to evaluate the outcomes from a clinically applicable environment. Unfortunately, cytotoxicity was reported from EBFCs, especially from oxygen sensitive enzyme (such as GOD), due to the high dose of H_2_O_2_ produced as a byproduct, leading to concerns about safety^24,25^. Therefore, a low level of current density for EBFCs, producing a sub-lethal concentration of H_2_O_2_ as a byproduct, has been utilized for generating electricity to stimulate cells instead of an electricity generator for the device. For example, muscle precursor cells and cardiomyocytes were favorably differentiated and functionally activated in the GOD system with a low current density (~14 nA/cm^2^) and relatively high current density (~2 µA/cm^2^); over tens of µA/cm^2^ adversely affect biological activities^26,27^. To achieve the high current density of EBFCs without safety concerns, GDH has been suggested as a replacement for H_2_O_2_ generating EBFCs, but a detailed investigation to reveal the biological safety and performance of GDH, according to our best knowledge, has not performed for comparison to that of GOD as a H_2_O_2_-producing EBFC counterpart.

Herein, we employed two-dimensionally screen-printed carbon electrodes to prepare GDH- and GOD-based EBFCs, which included osmium redox polymer complexes as a mediator and BOD as a cathode. After the electrochemical characterization of the EBFC systems, the GDH-based EBFCs were applied to two different cells, human dermal fibroblasts as a major resident cell type for tissue organization and Raw 264.7 cells for the inflammatory response^28–30^, to investigate the cytotoxicity, cell migration effects, and cellular inflammatory response of the byproducts and electrical cues (Fig. S1). Simultaneously, GOD-based EBFC revealing comparable electrochemical performance was designed as a counterpart and investigated for its biomedical application. To the best of our knowledge, this is the first attempt to design two different glucose-reactive EBFC systems and perform a comparison study to investigate its potential as a safe device to generate electricity for a body-implantable platform or even cell culture. These experiments suggest that GDH, with its cytocompatibility and cell-stimulating effects, is more promising than GOD for use in an implantable EBFC system to generate electricity for biomedical applications.

## Experimental Section

### Enzyme and chemical reagents

Glucose dehydrogenase (GDH; flavin adenine dinucleotide (FAD)-dependent) from *Aspergillus oryzae* was purchased from Toyobo Enzyme, Inc. (Japan). The glucose oxidase (GOD; FAD-dependent) from *Aspergillus niger* was purchased from Amano Enzyme, Inc. (Japan). The activity of the anode enzymes was certificated by the company (GDH = 584 U/mg, GOD = 243 U/mg). Bilirubin oxidase (BOD; 25 U/mg) from *Myrothecium verrucaria*, poly(ethylene glycol) diglycidyl ether (PEGDGE), sodium hydrosulfite, 1-vinylimidazole, acrylamide, *N*,*N*,*N*′,*N*′-tetramethyl ethylenediamine, and ammonium persulfate were purchased from Sigma-Aldrich Co. (Milwaukee, WI, USA). All other solutions including phosphate-buffered saline (PBS) were prepared using deionized Milli-Q water (DW; Millipore, Japan). PAA-PVI-[Os(dmo-bpy)_2_Cl]^+/2+^ (−0.012 V vs. Ag/AgCl) and PAA-PVI-[Os(dCl-bpy)_2_Cl]^+/2+^ (0.355 V vs. Ag/AgCl) as anode and cathode mediators were synthesized by modifying previously described methods (Fig. S2A)^31^.

### Fabrication of anode and cathode electrodes

**Figure 1:**
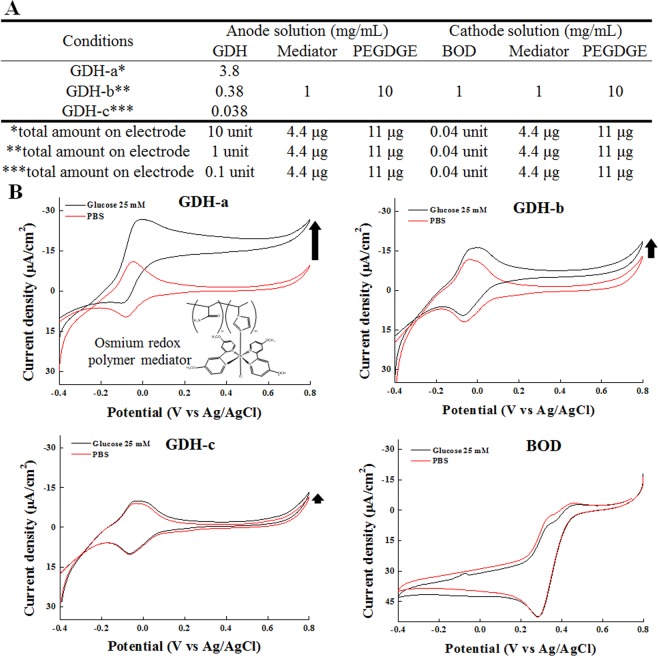
The components of the EBFCs (**A**) and their electrochemical characteristics (**B**–**E**). The cyclic voltammograms of (**B**) GDH-a, (**C**) GDH-b, (**D**) GDH-c, and (**E**) BOD concentrations with 25 mM glucose (black line) and PBS (red line) under ambient air after 5 min inject of compressed air before experiments with a scan rate of 0.01 V/sec at 25 °C.

Ten microliters of stock solution including GDH enzyme, mediator (PAA-PVI-[Os(dmo-bpy)_2_Cl]^+/2+^), and PEGDGE (4:4:1 v/v%) was cast onto each screen-printed carbon electrode (SPCE, 2 kΩ resistor) (Fig. S2B). In case of GOD anode, 0.926 mg/mL of GOD enzyme (GOD-b) with the same amounts of redox mediator and crosslinker from GDH anode was deposited. Figure 1A shows the total amounts of the enzymes, redox mediator, and crosslinker, respectively. All enzymes were prepared in 1× PBS. In addition, all mediators and PEGDGE were dissolved in DW. All cathode electrodes were loaded in the same condition by using stock solution containing BOD (10 U/mL in PBS), PAA-PVI-[Os(dCl-bpy)_2_Cl]^+/2+^ (0.5 mg/mL in DW), and PEGDGE (10.0 mg/mL in DW) in a 4:4:1 volume ratio (v/v%).

### Electrochemical characterization of EBFCs

All anode and cathode electrodes of the EBFC were measured by a CHI 660B potentiostat/galvanostat (Austin, TX, USA) with a Ag/AgCl reference electrode, −0.4 to 0.8 V scan range and 0.01 V/sec scan rate at 25 °C. Additionally, the power density was collected under a 2 kohm load in 1X PBS solution with 25 mM glucose at determined time points while EBFC was placed in the cell culture medium (supplemented DMEM) for 2 days. For measuring electrical performance, compressed air was injected at a speed of 5000 standard cubic centimeters per minute for 5 min before experiments if not mentioned otherwise. Nitrogen (99.9%) or oxygen (99.9%) gas was injected before experiment.

### Cytocompatibility of EBFC

Human dermal fibroblasts (HDFs) were chosen for a model cell line to investigate the cytocompatibility of the EBFC^32^ and were cultured in appropriate cell media. After one day of culturing HDFs, different types of prepared EBFCs or hydrogen peroxide (0~1500 µM) were inserted (or added) onto the plate and incubated for 24 hours to measure the cytotoxicity according to the manufacturers’ procedures^33–35^. The details of the protocols were described in the supplementary data. The LDH released was measured using a LDH Cytotoxicity Assay Kit (Thermo Fisher, USA) following the manufacturer’s protocols^36^. Briefly, 50 μL of the supernatant after 24 h co-culturing with EBFC was transferred to new 96-well plates, and then 50 μL of LDH assay solution was added. After being incubated for 30 min in a dark room, the total LDH released was then measured at 490 nm. The data are presented compared to the control as percentages.

### Hydrogen peroxide generation

The hydrogen peroxide generated from the EBFCs was measured by an assay kit (Cell Biolabs, Inc.; OxiSelect^Tm^ *In Vitro* ROS/RNS Assay Kit (Green Fluorescence))^37^. EBFC was inserted in PBS with 25 mM glucose, and H_2_O_2_ was measured after 24 hours by the absorbance at 450 nm using 96-well plates.

### Cell migration with EBFCs

The cells were seeded (2.0 × 10^4^) in 12-well plates and cultured for 2 days until reaching confluence. A scratch migration assay was performed with 10 µg/mL mitomycin C (Sigma) pretreatment to inhibit cell growth during the migration study^38^. A 200-μL pipette tip was used to scratch the cells, and an EBFC was inserted into each well. The scratched area was visualized at various times (0, 6, and 12 hours) under a microscope (IX-71, Olympus, Shinjuku, Tokyo, Japan), and the migrated cells were quantified by ImageJ (NIH) according to previously described procedures^29^.

### Multiplex screening assay for inflammatory cytokine release

Inflammatory cytokines in the supernatants from RAW 264.7 murine macrophages with EBFCs were analyzed by a magnetic Luminex screening assay kit (R&D Systems, Minneapolis, MN, USA) according to the manufacturer’s protocol^30,39^. The details of the protocols are provided in the supplementary data.

### Statistics

All data are presented as the representative mean ± one standard deviation after at least triplicate experimental sets. The one-way analysis of variance (ANOVA), followed by Tukey’s post hoc test, was performed at a significance level of P < 0.05.

## Results and Discussion

### Electrical performance of EBFCs

**Figure 2:**
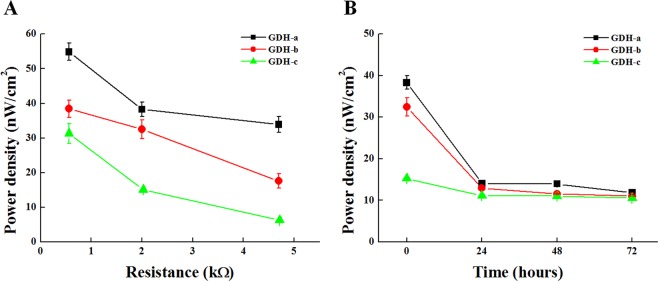
The power density at different (**A**) resistances and (**B**) over time for GDH-a, (black line), GDH-b (red line), and GDH-c (green line) at initial time for an initial resistance of 2 kΩ.

The components of the fabricated EBFCs are indicated in Fig. 1A. The EBFCs were designed to have various electrochemical characteristics depending on the amount of GDH enzyme. Figure 1B shows the individual cathode and anode electrode characteristics used in the EBFC, as investigated by cyclic voltammetry (CV) with Ag/AgCl as the reference electrode and a Pt wire as the counter electrode with/without glucose solution (25 mM). GDH-a showed the highest catalytic current (−26.9 µA/cm^2^ at 0.8 V vs. Ag/AgCl) with glucose compared to PBS (−9.73 μA/cm^2^ at 0.8 V vs. Ag/AgCl) due to the high amount of GDH, while GDH-c showed decreased catalytic currents (−13.4 μA/cm^2^ at 0.8 V vs. Ag/AgCl) compared with the GDH-a (−26.9 μA/cm^2^) and -b (−18.7 μA/cm^2^) condition due to the low amount of GDH. These results mean that GDH-a, -b, and -c utilize glucose to generate electrical current in a GDH enzyme amount dependent manner. The full system of the EBFC with the anode and cathode was tested under 25 mM glucose under ambient air for measuring the electrical performance by the I-t curve and open-circuit potential in cell culture system under ~20% oxygen and ~5% CO_2_, similar oxygen level to ambient air, which could affect biological functions. First, the power densities of the EBFCs were investigated with various resistance (0.56, 2.0 and 4.7 kΩ) loads (Fig. 2A) because, depending on the resistance applied in the EBFC system, the power density can vary. At 0.56 kΩ, the power density values of GDH-a, -b, and -c were 54.9 ± 2.53, 38.5 ± 2.5, and 31.3 ± 2.8 nW/cm^2^, respectively. At 2.0 kΩ, GDH-a, -b, and -c were 38.3 ± 2.1, 32.5 ± 2.7, and 15.1 ± 0.7 nW/cm^2^, while at 4.7 kΩ, GDH-a, -b, and -c showed compromised power densities (33.9 ± 2.3, 17.6 ± 2.1, and 6.27 ± 0.82 nW/cm^2^). When electrical performance of EBFC (GDH-a as an representative) was measured under nitrogen or oxygen condition, comparable current density was measured regardless of gas used due to innate characteristics of oxygen-insensitive enzyme (GDH), unlike oxygen-sensitive enzymes (i.e.GOD)^25,40^, and limitation of oxygen supply to BOD for increasing power output (Fig. S3A)^41^.

The EBFC with a 2.0 kΩ load was chosen for measuring the stability over time because the SPCB electrode (width = 0.2 cm and length = 5.2 cm) for the EBFC system has ~2.0 ± 0.5 kΩ loads. Under a full system consisting of the anode (GDH), cathode (BOD) and SPCB, GDH-a, -b and -c revealed power densities of 38.3 ± 2.1, 32.5 ± 2.7, and 15.1 ± 0.7 nW/cm^2^, respectively, depending on the loaded amounts of GDH enzyme in the EBFC system. Unfortunately, all conditions of EBFCs showed only a short-term stability of generating electrical cues, revealing that less than 20% of the power density remained after 24 hr incubation in 25 mM glucose (in 1X PBS) compared to the initial power density (Fig. 2B). When current density was measured during continuous operation up to 24 hr, current density was very initially (~1.2 minutes) different among groups (initial value; 11, 7.8, and 7.1 µA/cm^2^ respectively from GDH-A, -B and -C) and similarly maintained afterward (Fig. S3D). The main reason of short-time stability above is possibly due to the degradation of enzyme immobilized on electrode^1,42^. Many attempts have been performed to increase enzyme stability with the help of continuous reactant concentration (i.e. glucose), enzyme immobilization or enhanced reaction area by 3D structure^43–45^, which will be utilized further for enhancing or prolonging biological functions of EBFC. Within the limitation of the above short-term stability of generating electrical cues, EBFCs were applied for culturing cells in media for up to 24 hrs (Fig. S1), because according to the other literatures, short time (10 s ~ 2 minutes) electrical cues possibly showed significant biological performance^46,47^. For longer period of *in vitro* or *in vivo* biological experiments, repeated EBFC implantation strategy or developing EBFC with longer stability are possibly suggested, which will be further studied^42,43,48^. GOD-based EBFCs using the same enzyme units were fabricated as a counterpart for GDH-b with the same enzyme units (0.1) on the EBFC system. The polarization curves, including the power density and open-circuit potential (OCP), of the anode and cathode electrodes were obtained for comparison, as shown in Fig. S4A and B. The maximum power density and OCP from GDH-b and GOD-b are 0.36 V and 10.88 µW/cm^2^ and 0.38 V and 11.22 µW/cm^2^, respectively.

### Cell viability with EBFCs

Cytocompatibility tests of biomaterials implanted for a short period have generally been performed to investigate the initial biocompatibility^49^. Here, the HDF viability was investigated for 1 day after being incubated with two different EBFC types (GDH and GOD) and various enzyme concentrations. HDFs cultured with SPCE or paraformaldehyde (PFA) were used as negative and positive controls, respectively, for the cytotoxicity test because all EBFCs were deposited on the SPCE before being implanted in the culture media. As a preliminary study, the GOD-b based EBFC among the GOD-based EBFCs was utilized to check the cell viability, revealing severe cytotoxicity with a high generation of hydrogen peroxide (~1500 µM) above the lethal dose to human fibroblasts^50^ (Fig. S4C-F), confirmed by the absence of green-colored live cells in GOD-b and extremely low cell viability from GOD-b and its H_2_O_2_ equivalent (1000~1500 µM). The dead cells (red) in GOD-b were not visualized because the dead cells detached easily and were washed out during the PBS washing before the live/dead staining^51^. Hydrogen peroxide generation on the GOD-b can also be from the oxygen reduction by the relatively low redox potential of Os-polymer itself especially when Os-polymer has less value (*E*°′) than + 0.07 V *vs*. Ag./AgCl (PAA-PVI-[Os(dmo-bhy)_2_Cl]^+/2+^, −0.012 V)^52^. Thus, to reveal the mechanism how GOD based EBFC system generate hydrogen peroxide generation, future studies using other Os-polymers with high redox potential (>0.07 V) might be necessary. However, taken the results from GDH-b, deposited on anode with same type and amount of Os-polymer similar to GOD-b, based EBFC system that there was absence of hydrogen peroxide (Fig. S4E), the generation of hydrogen peroxide was possibly due to the nature property of GOD.

**Figure 3:**
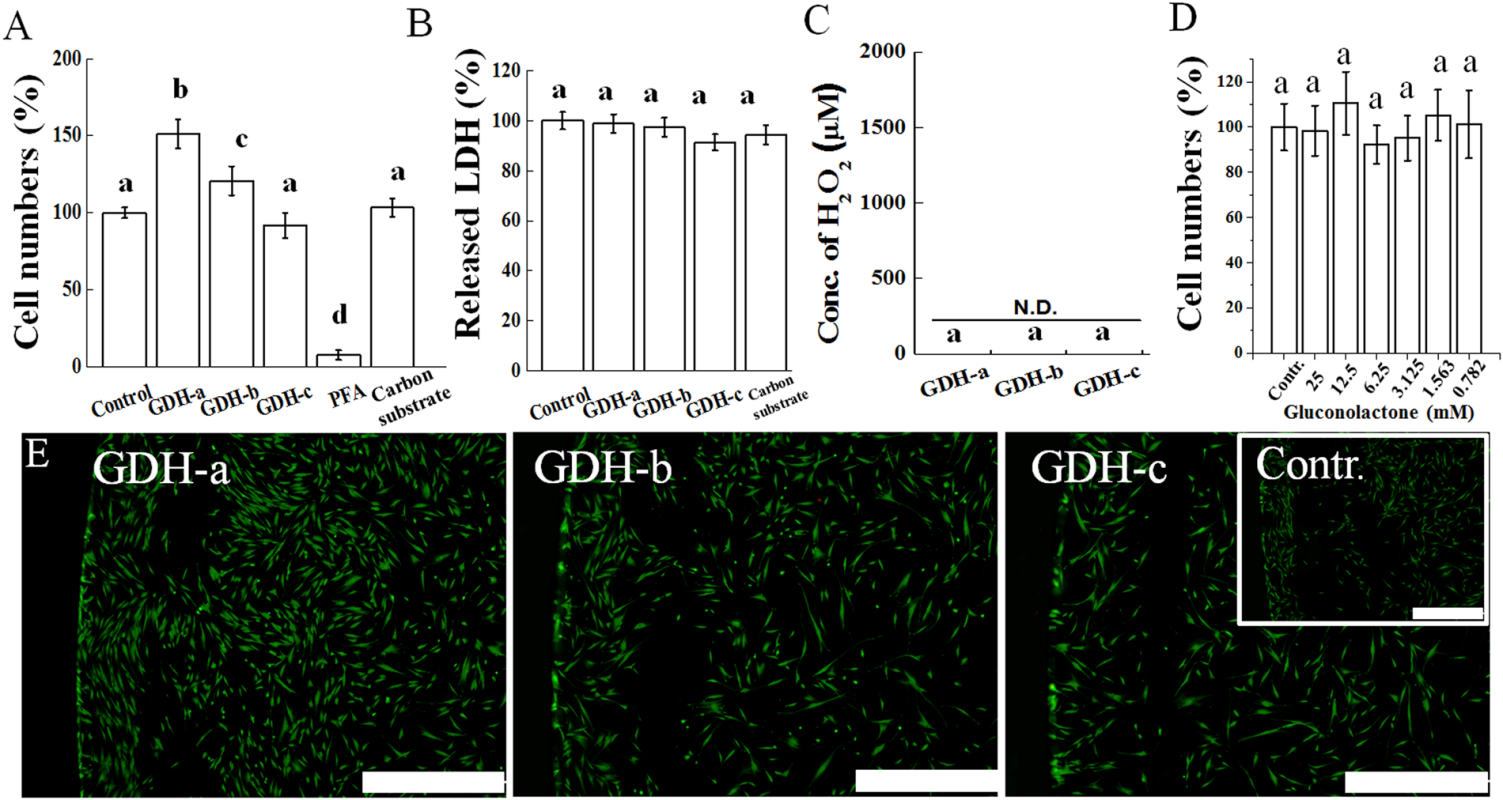
Human dermal fibroblast cytotoxicity of GDH-based EBFC for 1 day of culture. (**A**) Cell numbers and (**B**) released lactose lactate dehydrogenase due to cell damage. (**C**) Amounts of generated hydrogen peroxide. (**D**) Cell numbers depending on the gluconolactone concentration, which may be released from the EBFC as a byproduct. All assays were performed with 25 mM glucose, which was the same concentration as used in the cell culture condition. (**E**) Live (green) & dead (red) cells were visualized. An increase in cell viability (~150%) was observed with GDH without cell damage and hydrogen peroxide generation, which was confirmed by the live/dead imaging (P < 0.05). Different letters (i.e. a, b, c, d) without overlap indicate a significant difference between the conditions (P < 0.05). Scale bar is 1 mm.

In the case of the GDH-based EBFC, compared to the control, GDH-a and -b showed ~50% and ~20% increases in cell numbers, while all GOD groups showed significant decreases in cell viability to less than 10% (Fig. 3A, P < 0.05). The direct cell toxicity was checked by an assay kit of lactate dehydrogenase, released into the media when cells are damaged and used as a biomarker for cellular cytotoxicity, revealing no cytotoxicity from all GDH groups (Fig. 3B, P > 0.05). To investigate any release of H_2_O_2_ generated in culture media, after one day incubation of the EBFCs with 25 mM glucose, the H_2_O_2_ was measured, and there was no significantly detection in all GDH in accordance with the reaction formula when the GDH meets glucose (Fig. 3C, P > 0.05). One of the possible causes for the increased cell viability is the gluconolactone, which is a byproduct of the GDH-glucose reaction. When various amounts of gluconolactone up to the maximum concentration (25 mM) produced by the glucose (25 mM)-GDH reaction in media was applied to the HDFs, there was no significant cell viability change in the gluconolactone-treated groups (0.782~25 mM) compared to the control (P > 0.05, Fig. 3D). EBFC’s other components such as osmium redox polymer complexes and crosslinker (PEGDGE), which can be released into culture media, were proven not to affect the cell viability at the concentration used for this EBFC system^27^. Live/dead staining revealed the abundant presence of live cells in all GDH groups, confirming the above cell viability results (Fig. 3E). The GDH-a and -b groups showed more cells than the control, which can explain the increase in the cell viability. Taken together, the GDH-based EBFC system generates electricity without H_2_O_2_ and increases the cell numbers by electrical stimulation, while the GOD-based EBFC system revealed cytotoxicity due to the generated H_2_O_2_^53,54^. The change of the transmembrane potential of the cells via electrical stimulation can be a possible mechanism for the above biological phenomenon by various intracellular signaling pathways, such as mitogen-activated protein kinase/extracellular signal regulated kinases that are involved in the regulation of the cellular activity^55,56^.

A previous study also revealed the cytotoxicity of an CDH-based EBFC system due to the generation of H_2_O_2_ as a byproduct^24^, which can be compensated by an additional catalyst. However, there was inconsistency with other research revealing a certain cell survival (~50%) even at 10~12 μA/cm^2^ from the GOD. The difference in the EBFC systems connecting the anode and cathode could explain the inconsistency; previous studies implanted GOD and BOD separately in cell culture conditions as a non-connected EBFC system, and electrons, hydrogen ions and other byproducts flowed via the electrolyte, contributing to a limited (or lower) potential (<0.4 V)^26,27^. On the other hand, this study physically connected the anode (GOD-b) and cathode (BOD) by electroconductive carbon tape, which allowed electron movement so that the designed potential (0.4 V) would mimic the environment when EBFC is installed in a living body and touching tissue^21^. Then, the connected EBFCs can generate byproducts (i.e., H_2_O_2_) and hydrogen ions faster and in larger quantities than that from non-connected EBFCs, which can kill cells more effectively. As for other reasons, the different lethal thresholds against electricity depending on the cell type and cell number can explain the various cell sensitivities^57^.

### *In vitro* inflammation test with EBFCs

**Figure 4:**
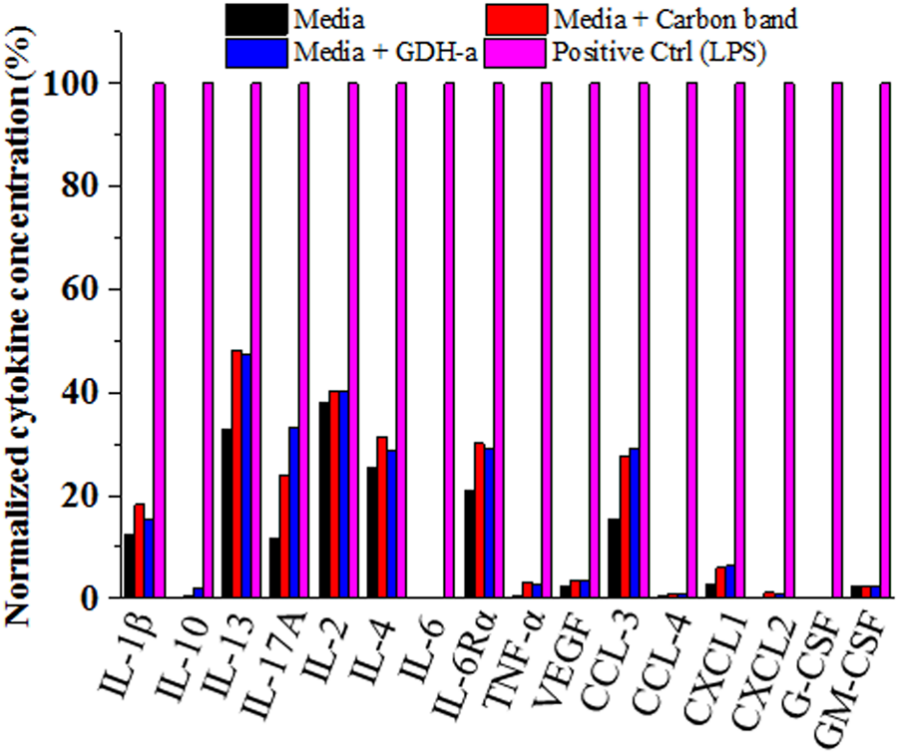
Inflammatory effects of GDH-based EBFC using immune cells (mouse macrophages, RAW cells 264.7). EBFCs were co-cultured with the non-cytotoxic GDH-b EBFCs as a representative, and the supernatant was collected for an inflammatory cytokine array. Generally, carbon tape substrate (SPCE) and GDH-based EBFC resulted in comparable inflammatory cytokine generations.

To investigate the possible adverse effects that occur when an EBFC is implanted in the body and contacts macrophages, thus regulating the tissue regeneration potential, RAW 264.7 cells, a representative macrophage cell line widely used for investigating the inflammatory effects of implanted biomaterials, were co-cultured with the non-cytotoxic GDH-b EBFCs, and the supernatant was collected for an inflammatory cytokine array. The concentration of most of the inflammatory cytokines in GDH-b was comparable to that of the controls (conditioned media from cells and cells + SPCE), and the absolute concentration of these cytokines was in the safe range and was less than the concentrations for severe inflammatory control from LPS treatment^58,59^ (Table S1 and Fig. 4). The levels of a few chemokines (i.e., chemokine (C-C motif) ligand, chemokine (C-X-C motif) ligand, and colony-stimulating factors) and biomolecules recruiting macrophages and other types of cells for certain purposes were more than 100 pg/mL higher in the GDH-a. However, those concentrations were lower than those of the positive inflammatory control (~30%) and were in a safe range. A detailed investigation of the inflammatory effects from the enhanced inflammatory cytokines will be necessary for further study. From the findings showing an increase in the cell viability of the culture without a significant inflammatory response in the GDH groups, cytocompatibility and a cell-stimulating effect from the GDH can be expected when GDH is implanted in the human body^58^.

### Migration of HDFs with EBFC

**Figure 5:**
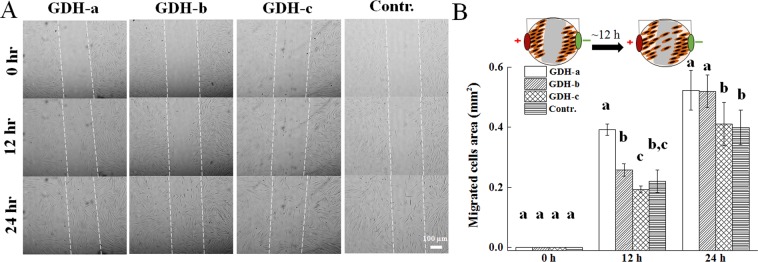
Accelerated cell migration effect from EBFC for 24 hr co-culturing. The representative images of (**A**) the migration assay and (**B**) their quantification data. An increase in the cell migration (~90%) was observed with GDH-a compared to the control at 12 hr incubation, and accelerated migration was maintained for up to 24 hr (~25%). Different letters (i.e. a, b, c) without overlap for the same culture time indicate a significant difference between the conditions (P < 0.05).

HDF migration, that is, the movement behaviors of cells in a specific direction, is one of the critical characteristics for measuring the cellular motility, which is helpful for tissue or organ wound healing after device implantation^60^. Therefore, a cell migration study was performed by using a wound scratch assay, which is a well-known method to mimic an *in vivo* environment where an electrical device is implanted in the human body^61^. GDH groups were used for the migration study, but all GOD groups were excluded due to their severe cytotoxicity. After mitomycin C pretreatment for 2 hours to inhibit cell proliferation, the confluent HDFs were scratched with a pipette tip to create a gap (~350 µm) and allowed to migrate toward the scratched area for up to 24 hours after EBFC implantation in a cell culture plate with the three different current densities for GDH-a, GDH-b, and GDH-c (Fig. 5A). After an initial 12-h implantation, there were significant differences in the migrated cells in GDH-a (~90% increase) compared to the others (P < 0.05). After 24 hours of implantation, the high and middle current density groups (GDH-a and –b) had more cell migration (~25% more) than the group with a lower current densities (GDH-c) and control (P < 0.05). The quantification of the migrated cells revealed that GDH treatment significantly enhanced the cell migration in the following order: SPCE control and GDH-c < GDH-a and b at 24 hr (Fig. 5B, P < 0.05). These results showed that current densities from −13.4 ~−26.9 μA/cm^2^ more effectively promote cell migration than those from zero ~ −9.73 μA/cm^2^. The current densities used for the migration assay are within the safe range and do not result in tingling sensations or pain^62^. However, previous studies mentioned adverse differentiation or physiological functionalities at 10~12 µA/cm^2^ compared to that of 0.014~2 µA/cm^2^ ^27,26^, which might be due to the negative effect from the H_2_O_2_ produced by the GOD enzyme. Physiologically, injured skin has an electrical current density of -10 to -100 μA/cm^2^, which can accelerate skin tissue regeneration via electrical stimulation and support the enhanced cell migration phenomenon^63,64^. Therefore, the electrical cues generated by the GDH system are in the physiologically safe range and can be further utilized for tissue regeneration applications.

## Conclusion

**Figure 6:**
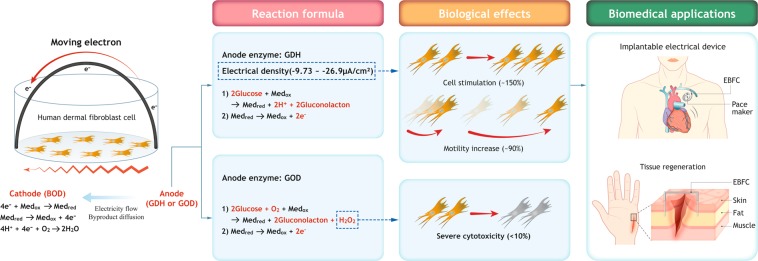
GDH can increase the cell viability and motility through electrical cues without severe cytotoxicity or an inflammatory response, while GOD induces severe cytotoxicity due to the production of a lethal concentration of hydrogen peroxide. Therefore, GDH is preferred for use in a glucose-reactive EBFC for implantable electrical devices and tissue regeneration.

In this study, we first investigated the HDF cellular activities of two different biomedical-applicable glucose-reactive EBFCs (GDH and GOD) with comparable electrical characteristics (Fig. 6). In the GDH groups, cytotoxicity was not detected, and cell stimulation (~150%) by electrical cues was even observed. The cellular inflammatory response of GDH was considered to be relatively low, but further investigation is necessary. However, the GOD-based EBFC revealed severe cytotoxicity (<10%) due to the production of a lethal concentration of H_2_O_2_ (~1500 μM) as a byproduct of the GOD. The HDF cellular motility, which is a key biological factor in tissue or organ wound healing after device implantation, was significantly enhanced in the GDH group (~90%), suggesting the promise of using GDH for biomedical applications in humans. Therefore, this research highlights the promising role of GDH-based EBFCs as biofuel cell systems to power biosensors or other types of implanted machines, as well as a biocompatible electricity-generating tool for mimicking/modulating the physiological/biological behaviors of cells for tissue regeneration^65,66^.

## Supplementary information

Supplementary information
