# Author Correction: Performance of a glucose-reactive enzyme-based biofuel cell system for biomedical applications

**DOI:** 10.1038/s41598-019-54377-7

**Published:** 2019-11-27

**Authors:** Won-Yong Jeon, Jung-Hwan Lee, Khandmaa Dashnyam, Young-Bong Choi, Tae-Hyun Kim, Hae-Hyoung Lee, Hae-Won Kim, Hyug-Han Kim

**Affiliations:** 1 Department of Chemistry, College of Natural Science, Dankook University, Chungnam Cheonan, 31116 Republic of Korea; 2 Institute of Tissue Regeneration Engineering (ITREN), Dankook University, Chungnam Cheonan, 31116 Republic of Korea; 3 Department of Nanobiomedical Science & BK21 PLUS NBM Global Research Center for Regenerative Medicine, Dankook University, Chungnam Cheonan, 31116 Republic of Korea; 4 UCL Eastman-Korea Dental Medicine Innovation Centre, Dankook University, Chungnam Cheonan, 31116 Republic of Korea; 5 Department of Biomaterials Science, College of Dentistry, Dankook University, Chungnam Cheonan, 31116 Republic of Korea

Correction to: *Scientific Reports* 10.1038/s41598-019-47392-1, published online 26 July 2019

In the original version of this Article, there were errors in the Affiliations which were incorrectly listed as ‘Department of Chemistry, College of Natural Science, East Lansing, USA,’ ‘Institute of Tissue Regeneration Engineering (ITREN), Chungnam, Republic of Korea.’, ‘Department of Nanobiomedical Science & BK21 PLUS NBM Global Research Center for Regenerative Medicine, Cairo, Egypt.’, ‘UCL Eastman-Korea Dental Medicine Innovation Centre, Chungnam, Republic of Korea.’, and ‘Department of Biomaterials Science, College of Dentistry, Dankook University, Chungnam, Cheonan, 31116, Republic of Korea.’, respectively.

The correct affiliations are listed below:

Affiliation 1

Department of Chemistry, College of Natural Science, Dankook University, Chungnam, Cheonan, 31116, Republic of Korea.

Affiliation 2

Institute of Tissue Regeneration Engineering (ITREN), Dankook University, Chungnam, Cheonan, 31116, Republic of Korea.

Affiliation 3

Department of Nanobiomedical Science & BK21 PLUS NBM Global Research Center for Regenerative Medicine, Dankook University, Chungnam, Cheonan, 31116, Republic of Korea.

Affiliation 4

UCL Eastman-Korea Dental Medicine Innovation Centre, Dankook University, Chungnam, Cheonan, 31116, Republic of Korea.

Affiliation 5

Department of Biomaterials Science, College of Dentistry, Dankook University, Chungnam, Cheonan, 31116, Republic of Korea.

These errors have now been corrected in the PDF and HTML versions of the article and in the Supplementary Information.

