# Supplementary material for: Performance of a glucose-reactive enzyme-based biofuel cell system for biomedical applications
Source: Sci Rep. 2019 Jul 26;9:10872. doi: 10.1038/s41598-019-47392-1 (PMC6659637; doi:10.1038/s41598-019-47392-1)
Supplement: Supplementary file 1 — Supplementary information [file 41598_2019_47392_MOESM1_ESM.docx]

**Performance of a glucose-reactive enzyme-based biofuel cell system for biomedical applications**

Won-Yong Jeon^1,2,4,†^, Jung-Hwan Lee^2,3,4,5†, *^, Khandmaa Dashnyam^2,3,4^, Young-Bong Choi^1^, Tae-Hyun Kim^2,3,4,5^, Hae-Hyoung Lee^2,4,5^, Hae-Won Kim^2,3,4,5*^, Hyug-Han Kim^1,*^

**Affiliations:**

^1^Department of Chemistry, College of Natural Science, Dankook University, Chungnam, Cheonan, 31116, Republic of Korea.

^2^Institute of Tissue Regeneration Engineering (ITREN), Dankook University, Chungnam, Cheonan, 31116, Republic of Korea.

^3^Department of Nanobiomedical Science & BK21 PLUS NBM Global Research Center for Regenerative Medicine, Dankook University, Chungnam, Cheonan, 31116, Republic of Korea.

^4^UCL Eastman-Korea Dental Medicine Innovation Centre, Dankook University, Chungnam, Cheonan, 31116, Republic of Korea

^5^Department of Biomaterials Science, College of Dentistry, Dankook University, Chungnam, Cheonan, 31116, Republic of Korea

^†^ W.-Y. Jeon ([powerwy84@gmail.com](mailto:powerwy84@gmail.com)), and J.-H. Lee ([ducious@gmail.com](mailto:ducious@gmail.com)) contributed equally to this paper as first authors.

^*^ Corresponding author. E-mail: [kimhw@dankook.ac.kr](mailto:kimhw@dankook.ac.kr) (H.-W. Kim; Tel.: +82-52-550-3082, Fax: +82-41-7840), [hankim@dankook.ac.kr](mailto:hankim@dankook.ac.kr) (H.-H. Kim; Tel.: +82-41-550-3435, Fax: +82-41-559-7860), and [ducious@gmail.com](mailto:ducious@gmail.com) (J.H. Lee, Jung-Hwan Lee; Tel.: +82-52-550-3082, Fax: +82-41-7840) contributed equally to this paper as corresponding authors.

Contact E-mails: [khandmaa@naver.com](mailto:khandmaa@naver.com) (K.D.), [chem0404@dankook.ac.kr](mailto:chem0404@dankook.ac.kr) (Y.-B.C.), [nerowolf@dankook.ac.kr](mailto:nerowolf@dankook.ac.kr) (K.-T.H.) and [haelee@dankook.ac.kr](mailto:haelee@dankook.ac.kr) (H.-H.L)

**Supplementary**

**Materials and methods**

**Cytocompatibility of EBFC**

Human dermal fibroblasts (HDFs) were chosen for a model cell line to investigate the cytocompatibility of EBFC [^31^](#_ENREF_31) and were cultured in DMEM (high glucose: 4,500 mg/L; 25 mM) supplemented with 10% [fetal bovine serum](https://en.wikipedia.org/wiki/Fetal_bovine_serum) (Gibco, Waltham, MA, USA) and 1% penicillin streptomycin (Gibco, Waltham, MA, USA). The HDFs (2.0 × 10^4^) were seeded in 12-well plates and cultured for one day at 37 °C and 5% CO_2_ in a humidified incubator. Different types of prepared EBFCs were inserted onto the plate and incubated for 24 or 48 hours to measure the cytotoxicity by the Cell Counting Kit-8 Assay (CCK-8; Dojindo Molecular Technologies, Inc.) and live/dead staining (Thermo Fisher, Waltham, MA, USA) according to the manufacturers’ procedures [^32^](#_ENREF_32)^,^[^57^](#_ENREF_57). Briefly, after 2 hours of incubation with CCK-8 solution (10%), 100 μL of medium was transferred to 96-well plates at determined times for absorbance measurements at 450 nm using a microplate reader (SpectraMax M2e, Molecular Devices, Sunnyvale, CA, USA). For live/dead staining, after rinsing with PBS, calcein AM and ethidium homodimer-1 were applied to detect live (green) and dead (red) cells, which were visualized under a fluorescence microscope (IX-71; Olympus, Tokyo, Japan) with a 4× lens [^32^](#_ENREF_32).

**Multiplex screening assay for inflammatory cytokine release**

The inflammatory cytokines in the supernatants from RAW 264.7 murine macrophages with EBFCs were analyzed by a magnetic Luminex screening assay kit (R&D Systems, Minneapolis, MN, USA) according to the manufacturer’s protocol [^29^](#_ENREF_29)^,^[^38^](#_ENREF_38). Briefly, 50,000 RAW 264.7 cells were seeded in each well of 12-well culture plates and supplemented with DMEM (10% FBS and 1% PS). The next day, this culture medium was replaced with 1 mL of DMEM, and EBFCs were inserted in each well. The supernatants were collected at 24 and 48 hours and used for the inflammatory cytokine analysis. The collected samples were added to a 96-well plate preconjugated with a panel of anti-cytokine antibodies covalently linked to unique polystyrene beads, and the reaction of the samples was then analyzed by a Luminex MAGPIX analyzer (R&D Systems). A cytokine array assay was independently duplicated, and the average of the results was calculated. RAW 264.7 cells without EBFC were used as negative controls, and 1 µg/mL LPS (lipopolysaccharide)-treated RAW 264.7 cells were used as positive inflammatory controls.

**sFigures**


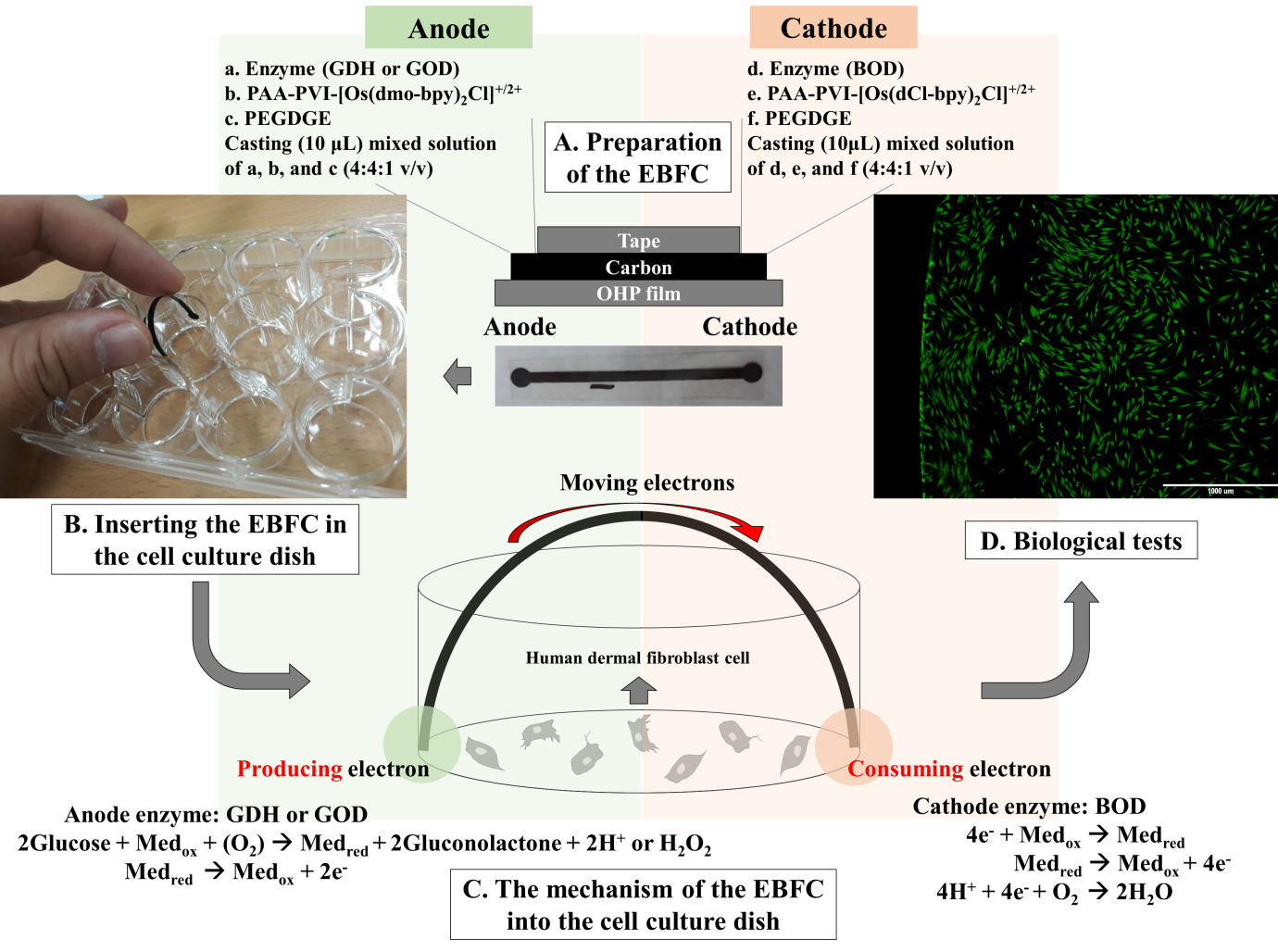


**Fig. S1**. Schematic illustration of in vitro experiments using HDFs. After an EBFC was cast on carbon tape, the EBFC was placed in the cell culture medium. Electricity and byproducts were generated and affected the biological activities of the HDFs.


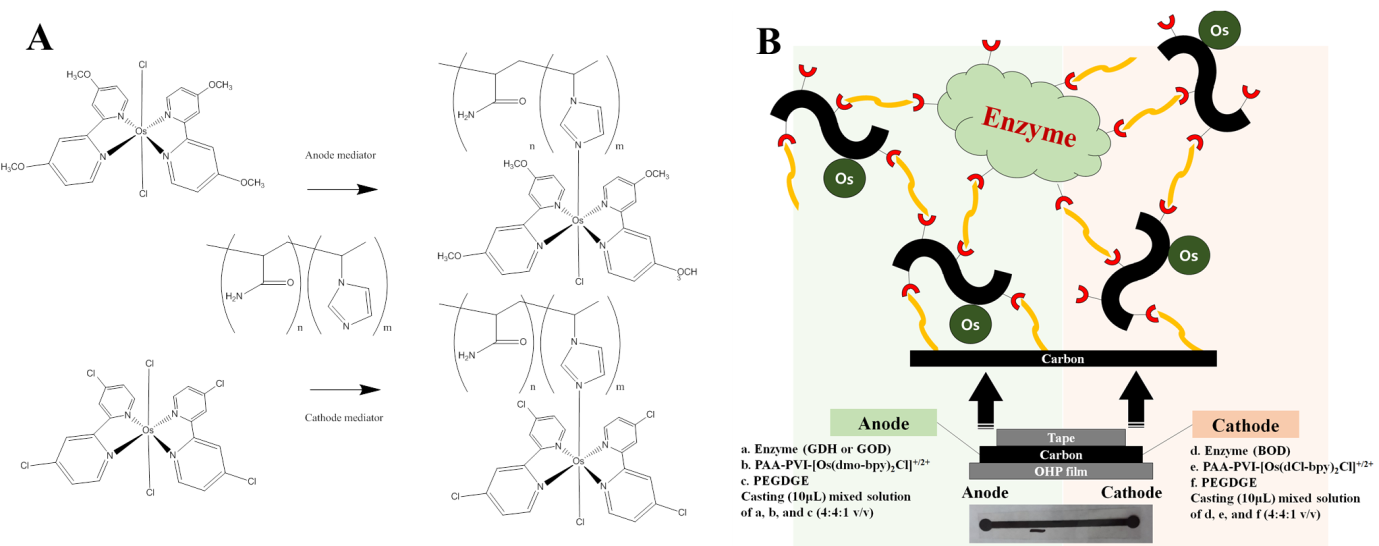
**Fig. S2**. Schematics of (A) the synthesis of the osmium redox polymer mediator and (B) the components of the anode and cathode electrodes of the EBFC.


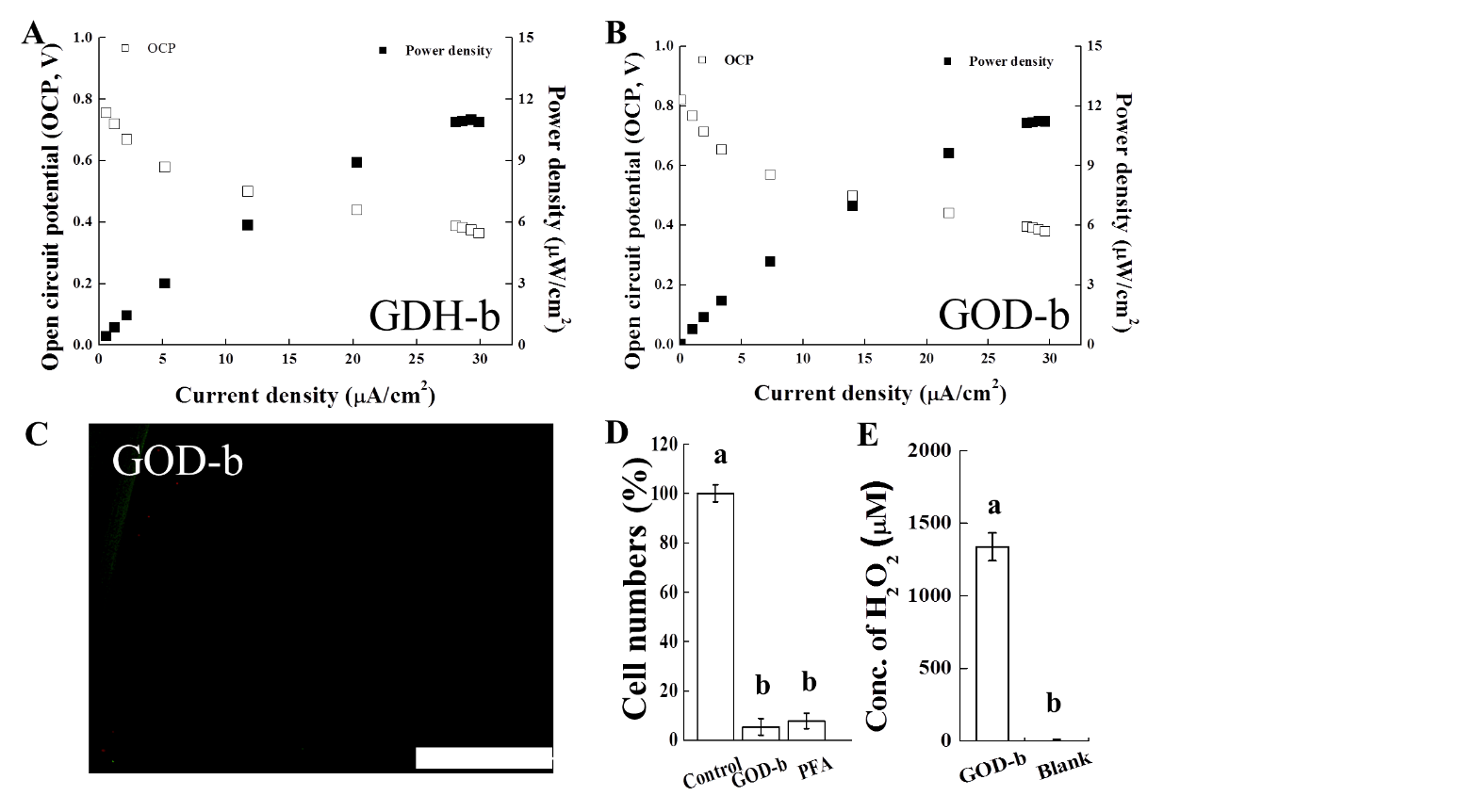


**Fig. S3.** Electrical and biological characteristics of two different EBFC systems. The polarization curves (A and B) of GDH-b and GOD-b were shown for representative comparison. (C) No live cells appeared in GOD-b, as confirmed by the (D) direct cell counting results and (E) lethal dose of hydrogen peroxide generated with 25 mM glucose.

**Table S1.** Results of the inflammatory cytokine array using RAW 264.7 macrophages (pg/mL)

|  | **N Ctrl** | **Band Ctrl** | **GDH-b** | **LPS** |
| --- | --- | --- | --- | --- |
| **IL-1β** | 40.0 | 36.5 | 52.9 | 289.8 |
| **IL-10** | 0.3 | 0.3 | 0.8 | 150.0 |
| **IL-13** | 41.0 | 33.1 | 48.2 | 100.5 |
| **IL-17A** | 3.2 | 2.1 | 4.3 | 17.9 |
| **IL-2** | 4.3 | 4.3 | 4.5 | 11.2 |
| **IL-4** | 25.2 | 30.7 | 38.2 | 121.3 |
| **IL-6** | 10.9 | 10.9 | 10.3 | 15028.3 |
| **IL-6Rα** | 68.4 | 138.6 | 198.3 | 659.1 |
| **TNF-α** | 5.9 | 7.3 | 37.4 | 1212.9 |
| **VEGF** | 1.4 | 1.3 | 1.8 | 53.2 |
| **CCL-3/MIP-1α** | 1287.7 | 1196.7 | 2113.4 | 7693.1 |
| **CCL-4/MIP-1β** | 721.9 | 595.8 | 1113.9 | 142259.1 |
| **CXCL1-/IP-10** | 52.0 | 50.6 | 110.4 | 1857.7 |
| **CXCL2/MIP-2** | 15.5 | 22.4 | 167.1 | 15399.0 |
| **G-CSF** | 4.8 | 9.5 | 18.1 | 20708.8 |
| **GM-CSF** | 6.6 | 6.4 | 6.3 | 261.4 |
